# Sulfide Generation by Dominant *Halanaerobium* Microorganisms in Hydraulically Fractured Shales

**DOI:** 10.1128/mSphereDirect.00257-17

**Published:** 2017-07-05

**Authors:** Anne E. Booker, Mikayla A. Borton, Rebecca A. Daly, Susan A. Welch, Carrie D. Nicora, David W. Hoyt, Travis Wilson, Samuel O. Purvine, Richard A. Wolfe, Shikha Sharma, Paula J. Mouser, David R. Cole, Mary S. Lipton, Kelly C. Wrighton, Michael J. Wilkins

**Affiliations:** a Department of Microbiology, The Ohio State University, Columbus, Ohio, USA; b School of Earth Sciences, The Ohio State University, Columbus, Ohio, USA; c Environmental Molecular Sciences Laboratory, Pacific Northwest National Laboratory, Richland, Washington, USA; d Department of Geology and Geography, West Virginia University, Morgantown, West Virginia, USA; e Department of Civil, Environmental and Geodetic Engineering, The Ohio State University, Columbus, Ohio, USA; University of Wisconsin-Madison; University of Michigan; Argonne National Laboratory

**Keywords:** *Halanaerobium*, shale, thiosulfate

## Abstract

Hydraulic fracturing of black shale formations has greatly increased United States oil and natural gas recovery. However, the accumulation of biomass in subsurface reservoirs and pipelines is detrimental because of possible well souring, microbially induced corrosion, and pore clogging. Temporal sampling of produced fluids from a well in the Utica Shale revealed the dominance of *Halanaerobium* strains within the *in situ* microbial community and the potential for these microorganisms to catalyze thiosulfate-dependent sulfidogenesis. From these field data, we investigated biogenic sulfide production catalyzed by a *Halanaerobium* strain isolated from the produced fluids using proteogenomics and laboratory growth experiments. Analysis of *Halanaerobium* isolate genomes and reconstructed genomes from metagenomic data sets revealed the conserved presence of rhodanese-like proteins and anaerobic sulfite reductase complexes capable of converting thiosulfate to sulfide. Shotgun proteomics measurements using a *Halanaerobium* isolate verified that these proteins were more abundant when thiosulfate was present in the growth medium, and culture-based assays identified thiosulfate-dependent sulfide production by the same isolate. Increased production of sulfide and organic acids during the stationary growth phase suggests that fermentative *Halanaerobium* uses thiosulfate to remove excess reductant. These findings emphasize the potential detrimental effects that could arise from thiosulfate-reducing microorganisms in hydraulically fractured shales, which are undetected by current industry-wide corrosion diagnostics.

**IMPORTANCE** Although thousands of wells in deep shale formations across the United States have been hydraulically fractured for oil and gas recovery, the impact of microbial metabolism within these environments is poorly understood. Our research demonstrates that dominant microbial populations in these subsurface ecosystems contain the conserved capacity for the reduction of thiosulfate to sulfide and that this process is likely occurring in the environment. Sulfide generation (also known as “souring”) is considered deleterious in the oil and gas industry because of both toxicity issues and impacts on corrosion of the subsurface infrastructure. Critically, the capacity for sulfide generation via reduction of sulfate was not detected in our data sets. Given that current industry wellhead tests for sulfidogenesis target canonical sulfate-reducing microorganisms, these data suggest that new approaches to the detection of sulfide-producing microorganisms may be necessary.

## Abstract (precis)

Although thousands of wells in deep shale formations across the United States have been hydraulically fractured for oil and gas recovery, the impact of microbial metabolism within these environments is poorly understood. Our research demonstrates that dominant microbial populations in these subsurface ecosystems contain the conserved capacity for the reduction of thiosulfate to sulfide and that this process is likely occurring in the environment. Sulfide generation (also known as “souring”) is considered deleterious in the oil and gas industry because of both toxicity issues and impacts on corrosion of the subsurface infrastructure. Critically, the capacity for sulfide generation via reduction of sulfate was not detected in our data sets. Given that current industry wellhead tests for sulfidogenesis target canonical sulfate-reducing microorganisms, these data suggest that new approaches to the detection of sulfide-producing microorganisms may be necessary.

## INTRODUCTION

Hydrocarbon-rich black shales underlie much of the continental United States and, through the development and application of horizontal drilling and hydraulic fracturing, contain economically recoverable quantities of natural gas and oil (1). Hydraulic fracturing involves the high-pressure injection of water, chemical additives, and proppant (usually sand) into a shale formation, generating fracture networks that release oil and gas to be recovered at the surface (2). Despite the application of biocides during the hydraulic fracturing process, a series of studies have revealed that microorganisms colonize these newly fractured shales and persist over extended periods of time (>300 days) (3–6). Such biomass accumulations in pipelines and reservoirs may be detrimental because of the potential for souring (production of H_2_S), microbially induced corrosion, and pore clogging by cells and biogenic gases (7, 8). Sulfide is a particular problem; toxicity associated with this compound poses health risks to workers and causes corrosion of steel infrastructure pipes by stimulating cathodic reactions that continuously leach protons from the metal (5). In offshore reservoirs, where sulfate intrusion into reservoirs (from seawater) is unavoidable, nitrate injections are frequently used to thermodynamically inhibit sulfide generation by sulfate-reducing bacteria (SRB) (6), while more recent studies have investigated perchlorate for the same role (6). While efforts are frequently made to ensure that low-sulfate fluids are used for hydraulic fracturing injections in terrestrial oil and gas reservoirs, little is known about sulfide generation in these systems.

Although microorganisms capable of catalyzing canonical sulfate reduction through the *dsrAB* enzyme complex have occasionally been detected in highly saline produced fluids from hydraulically fractured wells, they typically account for only a small percentage of the microbial population (7). Instead, previous studies have consistently identified fermentative *Halanaerobium* taxa as dominant colonizing microorganisms across different wells and shale plays (3, 5, 9, 10). In some instances, *Halanaerobium* strains may account for up to 99% of the relative abundance of the microbial community and are almost always detected as persisting key members in produced fluids (3, 4, 8–12). *Halanaerobium* genomic data, from both the reconstruction of genomes from metagenomic data sets and the sequencing of environmental isolates, have revealed the presence of multiple conserved rhodanese enzymes. Rhodanese enzymes are found in eukaryotic and prokaryotic cells and nonreductively cleave thiosulfate to elemental sulfur and sulfite (13). The sulfite produced from this reaction is subsequently reduced to sulfide, leading to the corrosion and toxicity issues described above (14).

Here we investigated the distribution of *Halanaerobium* sulfur-cycling genes across samples recovered from an Appalachian Basin shale play and confirmed the capacity of a *Halanaerobium* isolate from the Utica Shale formation to generate sulfide in the presence of thiosulfate via shotgun proteomics and chemical analyses. The Utica Shale formation was deposited approximately 443 million years ago and exists 2,000 m below the surface in southeastern Ohio near Flushing. Geochemical measurements of produced fluids from this site identified the presence of thiosulfate, while sulfur isotope measurements of the same samples were diagnostic of microbial reduction of oxidized sulfur compounds. These findings suggest that current industry monitoring centered on sulfate-mediated sulfidogenesis would not account for *Halanaerobium* sulfide generation, with long-term ramifications in hydraulically fractured environments.

## RESULTS

**FIG 1:**
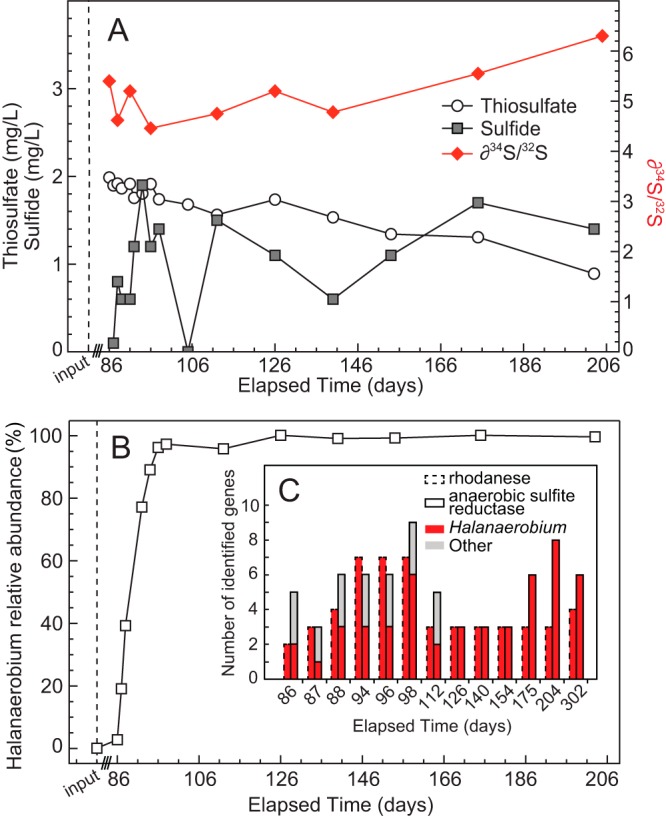
(A) Geochemical measurements of sulfur species from produced waters (~120-day time series) in Utica Shale. Sulfur isotope measurements were used to measure the reduction of oxidized sulfur species (sulfate and sulfonate from thiosulfate). The dashed line indicates down-hole injection of input fluids. Following the hydraulic fracturing process, the well was sealed for 86 days, during which no sampling could occur. (B) Changes in the relative abundance of *Halanaerobium* microorganisms in the period after hydraulic fracturing of the well. The dashed line indicates relative *Halanaerobium* abundance in input fluids prior to down-hole injection. (C) Number of reconstructed genes linked to thiosulfate transformations from metagenomic data sets collected during the monitoring period. Red indicates genes linked to *Halanaerobium*.

A temporal series of produced water samples were collected from a hydraulically fractured well in the Utica Shale in eastern Ohio for over 200 days after hydraulic fracturing. Following the fracturing process, the well was sealed (shut in) for 86 days, prohibiting the collection of fluid samples over this period. The well was fractured with lake water rich in oxidized inorganic sulfur species, as evidenced by the high concentration of sulfate in input fluids (904 mg/liter) (see Fig. S1 in the supplemental material). Thiosulfate was initially present at lower concentrations (~2 mg/liter) in produced waters and was likely derived from both hydraulic fracturing chemical additives and the leaching of salts from shale during water-rock interactions (15) (Fig. S2). Both sulfate and thiosulfate concentrations in produced waters decreased over the ~100-day monitoring period (Fig. 1A; Fig. S1), while persistent low-level sulfide concentrations (~1 mg/liter) were detected in the same samples (Fig. 1A). Finally, a temporal increase in δ^34^S_oxidized_ (sulfate and sulfonate from thiosulfate) values was concurrently measured, indicative of the microbial reduction of oxidized sulfur species such as thiosulfate (Fig. 1A).

10.1128/mSphereDirect.00257-17.1 FIG S1 Geochemical measurements of sulfate and barium concentrations from produced waters (~120-day time series) in Utica Shale. The dashed line indicates input fluid down-hole injection. Following the hydraulic fracturing process, the well was sealed for 86 days, during which no sampling could occur. Download FIG S1, PDF file, 0.1 MB. Copyright © 2017 Booker et al. 2017 Booker et al. This content is distributed under the terms of the Creative Commons Attribution 4.0 International license.

10.1128/mSphereDirect.00257-17.2 FIG S2 Thiosulfate abiotically leached from shales. Shale chips were left in deionized water for 1 and 2 days, as well as 2 and 3 weeks, with the longest leach incubation lasting 7 months. Ion chromatography was used to quantify thiosulfate at the end of each incubation period. Download FIG S2, PDF file, 0.02 MB. Copyright © 2017 Booker et al. 2017 Booker et al. This content is distributed under the terms of the Creative Commons Attribution 4.0 International license.

DNA was extracted from the same input and produced water samples used for geochemical analyses and analyzed with shotgun metagenomic tools. Reconstructed 16S rRNA gene sequences (processed with EMIRGE) (16) indicated that members of the genus *Halanaerobium* accounted for nearly 100% of the terminal microbial community for over 100 days (Fig. 1B; Table S1). Given the presence of oxidized sulfur species in input fluids, metagenomic data sets were screened for functional genes capable of sulfur transformations. Despite the high initial concentrations of sulfate, many of the essential genes involved in dissimilatory sulfate reduction (e.g., dissimilatory sulfite reductase [*dsrAB*], ATP sulfurylase [*sat*], and APS phosphokinase [*apsAB*]) were not detected across the time points studied. While one subunit of PAPS reductase was present in the metagenomic data sets, we hypothesize that this is playing a role in sulfur assimilation in *Halanaerobium*. Instead, the only genes associated with reductive sulfur transformations across all of the produced fluid metagenomes were associated with thiosulfate reduction. We recovered multiple rhodanese genes that encode the capacity to reduce thiosulfate to sulfite and elemental sulfur and genes for anaerobic sulfite reductases (*asr*) that reduce sulfite to sulfide (10, 13, 14, 17) (Fig. 1C). Consistent with the dominance of *Halanaerobium* in this time series (Fig. 1B), 100% of the rhodanese genes (*n* = 52) and 71% of the anaerobic sulfite reductase genes (*n* = 69) recovered in this data set were assigned to *Halanaerobium* (Table S2). The remaining *asr* genes (29%) were assigned to *Thermoanaerobacter* over the first three time points, reflective of this organisms’ initial high relative abundance (~26% of the 16S rRNA genes over this period [days 85 to 88]). Subsequently, the relative abundance of *Thermoanaerobacter* bacteria dropped to <3% in the remainder of the samples. On the basis of our field metagenomic and sulfur isotope geochemistry, we hypothesized that *Halanaerobium* bacteria were likely contributing to the reduction of thiosulfate *in situ*, a finding supported by other studies (10, 13, 14, 17).

10.1128/mSphereDirect.00257-17.3 TABLE S1 Reconstructed 16S rRNA gene sequences identifying terminal microbial community members from produced fluids over the 100-day sampling period. 16S rRNA gene sequences were processed with EMIRGE. Download TABLE S1, XLSX file, 0.6 MB. Copyright © 2017 Booker et al. 2017 Booker et al. This content is distributed under the terms of the Creative Commons Attribution 4.0 International license.

10.1128/mSphereDirect.00257-17.4 TABLE S2 Results of a BLASTp search for sulfur-cycling genes from *Halanaerobium* isolates against metagenomic databases constructed from produced water samples. Download TABLE S2, XLSX file, 0.04 MB. Copyright © 2017 Booker et al. 2017 Booker et al. This content is distributed under the terms of the Creative Commons Attribution 4.0 International license.

**FIG 2:**
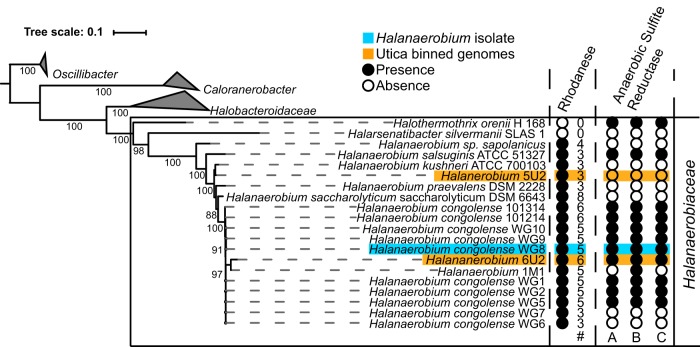
Phylogenetic placement of the environmental *Halanaerobium* isolate used in this study (WG8; highlighted in blue) relative to the reconstructed genomes of dominant *Halanaerobium* bacteria in Utica Shale-produced water samples (highlighted in orange). The eight Utica Shale *Halanaerobium* isolates are WG1, WG2, and WG5 to WG10. The numbers of rhodanese-encoding genes and anaerobic sulfite reductase subunits in each genome are illustrated to the right.

To support this observation, microbial laboratory enrichments were performed with synthetic saline medium and produced fluids (collected at 154 days postfracturing). These yielded eight *Halanaerobium* isolates that were closely related to *Halanaerobium* isolates identified via metagenomic analyses of produced waters (3) (Fig. 2). Genome sequencing of all eight isolates revealed between three and five copies of the gene encoding the rhodanese enzyme (Fig. 2) per genome. In addition, six of the eight isolates contained the three *asr* genes required for sulfide generation.

**FIG 3:**
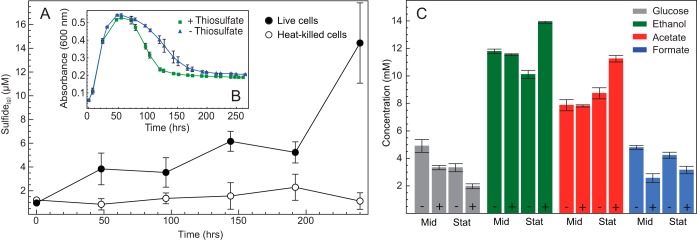
(A) Hydrogen sulfide production via thiosulfate reduction in live-cell incubations. (B) Growth curves of *Halanaerobium* WG8 in the presence or absence of thiosulfate. The calculated growth rates were 0.20 and 0.21 h^−1^, respectively. (C) Changes in the concentrations of glucose and major *Halanaerobium* fermentation products when bacteria are cultured in the presence (+) or absence (−) of thiosulfate at both mid-log (Mid) and stationary (Stat) growth phases. In all cases, error bars represent the standard deviation of the mean of triplicate biological replicates.

One *Halanaerobium* isolate (strain WG8)—containing five rhodanese genes and all three *asr* genes (Fig. 2)—was incubated in the presence or absence of exogenous thiosulfate. During 10-day incubations, the thiosulfate concentrations in live-cell cultures decreased by ~65 µM (data not shown). Concurrently, approximately six times as much H_2_S was generated in live cultures containing thiosulfate as in heat-killed controls (Fig. 3A), with the greatest sulfide production once *Halanaerobium* WG8 reached the stationary growth phase. The presence of thiosulfate had no statistically significant effect on exponential-phase growth rates (Fig. 3B).

Concurrent with sulfide generation, the greatest differences in the concentrations of major fermentation products were observed in stationary-phase cultures incubated in the presence or absence of thiosulfate. In the presence of thiosulfate, greater glucose consumption was coupled with consistent increases in acetate and ethanol concentrations (29 and 36%, respectively) and a decrease in formate concentrations (25%) relative to thiosulfate-free conditions during stationary phase (Fig. 3C). Notably, differences in fermentation product concentrations were less pronounced in mid-log-phase cultures, with only formate decreasing by approximately 45% in thiosulfate-amended cultures. Although no significant changes were detected in other common fermentation products, e.g., propionate and butyrate, concentrations of alanine were consistently higher in cultures grown with thiosulfate (Table S4).

**FIG 4:**
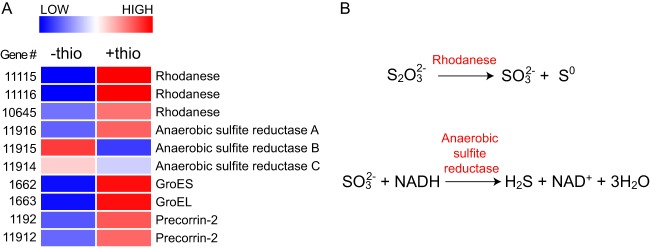
(A) Relative abundances of key proteins identified via shotgun proteomics that are implicated in sulfur transformations by *Halanaerobium* bacteria grown in the presence (+thio) or absence (−thio) of thiosulfate. (B) Reactions catalyzed by the rhodanese and anaerobic sulfite reductase enzymes.

To assess the cellular mechanisms of thiosulfate reduction, shotgun proteomic techniques were applied to biomass grown with or without exogenous thiosulfate in laboratory cultures. These tools revealed greater abundances of three rhodanese enzymes and one of three anaerobic sulfite reductase subunits (AsrA) in exponentially growing *Halanaerobium* bacteria with thiosulfate, indicating an active role for these proteins in sulfur transformations (14, 17) (Fig. 4; Table S3). Conversely, AsrB was present at greater abundances in the absence of thiosulfate. Given that other studies have reported the activity of Asr in the absence of thiosulfate and associated AsrB with the oxidation of NADH, the presence of this protein in *Halanaerobium* bacteria may be linked to the recycling of reducing equivalents from fermentative growth (18). In addition to rhodanese, chaperonin proteins GroES and GroEL and precorrin-2 dehydrogenase/sirohydrochlorin ferrochelatase were found in greater abundances in these same extracts. The GroES and GroEL proteins have been shown to promote the correct folding of mitochondrial rhodanese when cloned into Escherichia coli (19), while precorrin-2 dehydrogenase/sirohydrochlorin ferrochelatase is a bifunctional enzyme that catalyzes the conversion of precorrin-2 into siroheme, a prosthetic group required by anaerobic sulfite reductase (20).

10.1128/mSphereDirect.00257-17.5 TABLE S3 Proteomic results from *Halanaerobium* bacteria grown in the presence or absence of thiosulfate during laboratory incubations. Download TABLE S3, XLSX file, 0.04 MB. Copyright © 2017 Booker et al. 2017 Booker et al. This content is distributed under the terms of the Creative Commons Attribution 4.0 International license.

## DISCUSSION

Because of concerns associated with sulfidogenesis and scale formation, input waters rich in oxidized sulfur species are typically not used in hydraulic fracturing processes. However, in some instances where water is impacted by acid mine drainage (e.g., regions within the Appalachian Basin), the use of such fluids is sometimes unavoidable. Our temporal series of produced water samples was obtained from one such well targeting the Utica Shale in southeastern Ohio. Given the high concentrations of sulfate in initial produced water samples, it might be expected that SRB would dominate in this ecosystem. However, no evidence of SRB was detected in high-resolution metagenomic data sets and sulfate depletion in produced water samples was likely tied to the precipitation of a sulfate scale (e.g., barite) in the subsurface (Fig. S1). Scale formation in both conventional and unconventional reservoirs is a common issue where subsurface fluids contain high concentrations of total dissolved solids (TDS) (21). High TDS values (i.e., salinity) of up to ~200 g/liter have been reported in produced waters from shale plays in the Appalachian Basin (3, 11) and may additionally account for the absence of SRB within this system. Although SRB growth has been reported at salt concentrations of up to 270 g/liter (22), optimal salinity ranges are typically between 60 and 100 g/liter for halophilic sulfate reducers (23). SRB rely on the production of osmoprotectants to persist under saline conditions, and energetic yields from sulfate reduction (coupled with the need to synthesize osmoprotectants) may be insufficient to compete with fermentative *Halanaerobium* taxa under the high-salinity conditions characteristic of fractured shales in the Appalachian Basin (23).

Under such conditions, sulfide may instead be generated via the reduction of thiosulfate species, which in produced waters are likely derived from both hydraulic fracturing chemical additives (24) and the leaching of salts from the shale matrix during water-rock interactions (15) (Fig. S2). The leaching of thiosulfate from the shale matrix itself may represent a longer-term source of electron acceptor to support microbial metabolism once compounds from chemical additives have been depleted. However, this leaching (and subsequent consumption) of thiosulfate represents a cryptic cycle in the subsurface and limits our ability to assess the true extent of thiosulfate transformations over the monitoring period. The inherent reactivity of sulfide species poses similar challenges for quantitatively linking thiosulfate reduction with the accumulation of sulfide in produced waters. Regardless, our measurements did reveal overall decreases in thiosulfate concentrations over the sample collection period, coupled with the presence of persistent sulfide in produced waters. Sulfur isotope measurements in produced water samples provided further evidence of the microbial reduction of oxidized sulfur species (sulfonate from thiosulfate). In the absence of sulfate-reducing microorganisms across the sampled time points and the likely depletion of sulfate via abiotic scale formation, the temporal enrichment of ^34^S/^32^S in produced fluids suggests that thiosulfate reduction (catalyzed via *Halanaerobium* bacteria) was the major contributor to this signal. Multiple lines of evidence support our inference that *Halanaerobium* strains likely represent a major driver of thiosulfate-dependent sulfidogenesis in hydraulically fractured shale ecosystems. *Halanaerobium* bacteria were the dominant microbial taxa in this well for >100 days and contributed the genomic potential for the reduction of thiosulfate to sulfide across nearly all time points (Fig. 1B and C). The distribution of rhodanense-encoding and *asr* genes across both *Halanaerobium* genomes reconstructed from metagenomic data and microbial isolates indicates that the capacity for thiosulfate reduction is a relatively conserved trait within this genus.

Laboratory tests of a representative *Halanaerobium* isolate (WG8) from the same well indicated that live cultures generated sulfide when grown in a thiosulfate-amended medium in the laboratory, although no growth advantage was observed (Fig. 3B). The concentrations of microbially produced sulfide reported here are lower than those in a recent study that used a different *Halanaerobium* strain (9). In the absence of a genome sequence for the *Halanaerobium* bacteria used by Liang et al. (9), we hypothesize that strain level functional heterogeneity may play a role in these differences. The detection of small amounts of sulfide in heat-killed control experiments highlights the inherent reactivity of thiosulfate but also confirms the importance of live microbial cells in the generation of higher sulfide concentrations. Similar results showing no growth advantage in the presence of thiosulfate were previously obtained in experiments with Salmonella enterica subsp. *enterica* serovar Typhimurium, suggesting that the Asr enzyme complex plays a role in cell maintenance under nongrowth or electron acceptor-limiting conditions (14).

Proteomic analyses of *Halanaerobium* biomass collected during exponential phase revealed greater abundances of three rhodanese enzymes but only one Asr subunit in the presence of thiosulfate. We therefore suggest that anaerobic sulfite reduction by *Halanaerobium* is not coupled to growth but is instead used as a mechanism to dispose of fermentation reducing equivalents during the stationary growth phase. This inference is further supported by nuclear magnetic resonance (NMR) measurements of organic acid fermentation products, revealing significant concentration shifts during stationary phase between cultures grown in the presence or absence of exogenous thiosulfate. Thiosulfate utilization during fermentative growth has previously been investigated in Coprothermobacter platensis, with results demonstrating enhanced glucose utilization and alanine production in the presence of thiosulfate (25). The authors concluded that *C. platensis* utilizes thiosulfate as an electron sink, thus limiting the activity of hydrogenases and lessening hydrogen growth inhibition (25). A similar increase (2.5×) in alanine accumulation was observed when *Halanaerobium* bacteria were cultured in the presence of thiosulfate (Table S4), while proteomic measurements revealed lower abundances of hydrogenase proteins under these growth conditions (Table S3). Overall, these data suggest that *Halanaerobium* bacteria use thiosulfate as an electron sink, resulting in central metabolic changes that alter the relative ratios of fermentation end products. The accumulation of more organic acids (e.g., acetate) in addition to the production of sulfide in the presence of thiosulfate likely represents another mechanism via which *Halanaerobium* bacteria may stimulate corrosive processes in the deep subsurface infrastructure (26). That these changes are most pronounced during stationary phase suggests that the growth rate of *Halanaerobium* bacteria in shale environments may exert significant control over the extent of thiosulfate transformations, with greater potential for sulfidogenesis and organic acid production under the low-growth conditions commonly found in deep subsurface ecosystems (27).

10.1128/mSphereDirect.00257-17.6 TABLE S4 Concentrations of fermentation products generated by *Halanaerobium* bacteria growing on glucose in the presence or absence of thiosulfate. All values represent micromolar concentrations and were measured by ^1^H NMR. Download TABLE S4, XLSX file, 0.1 MB. Copyright © 2017 Booker et al. 2017 Booker et al. This content is distributed under the terms of the Creative Commons Attribution 4.0 International license.

To investigate the broader implications of rhodanese and Asr activity in subsurface environments, we screened for both sets of genes in the Joint Genome Institute (JGI) Integrated Microbial Genome (IMG) database. Results suggest that rhodanese-mediated thiosulfate transformations could play important roles in sulfur cycling across a broad range of ecosystems. Thiosulfate is frequently measured at low concentrations in subsurface systems, leading to the hypothesis that this substrate is rapidly consumed through disproportionation, oxidation to sulfate, or further reduction to sulfide (28). However, higher thiosulfate concentrations have been reported in salt marshes (micromolar concentrations in the hundreds) and have been attributed to high rates of pyrite oxidation (29). In deeper ecosystems, such as hydrothermal vents, the concentration of thiosulfate is not known, but pyrite oxidation is again thought to drive thiosulfate formation (30). The potential for the transformation of these thiosulfate pools is high because of the wide distribution of rhodanese enzymes across all domains of life (31). While rhodanese enzymes are present across multiple phyla (e.g., *Firmicutes*, *Proteobacteria*), rhodanese-encoding genes are more commonly found in tandem with the *asr* complex within the class *Clostridia* (includes *Halanaerobium*). Over 40% of the genomes within the class *Clostridia* (according to the IMG database) contained both the rhodanese-encoding and *asr* genes. The families representing this 40% were *Clostridiaceae*, *Eubacteriaceae*, *Lachnospiraceae*, *Peptococcaceae*, *Peptostreptococcaceae*, *Ruminococcaceae*, *Halanaerobiaceae*, *Halobacteroidaceae*, and *Thermoanaerobacteraceae*. There are 10 other families within *Clostridia* that did not contain organisms with both genes, but this could be attributed to their underrepresentation in the IMG database. Future studies could illuminate other rhodanese and *asr* partnerships within these families. However, the currently known *Clostridia* microorganisms that contain both genes were isolated across a range of ecosystems, including hydrothermal vents, deep sea water, deep terrestrial subsurface, oil fields, fresh water, and soil, with specific representatives including Carboxydothermus hydrogenoformans, *Orenia*, *Acetoanaerobium sticklandii*, and *Thermincola potens*. Where microorganisms lack the capability to further reduce sulfite (i.e., only produce rhodanese enzymes), these compounds can be utilized by nearby community members. Such metabolic “handoffs” have recently been shown via metagenomic analyses to be highly prevalent in some subsurface systems, with multiple microbial groups responsible for catalyzing complete reductive or oxidative transformations of redox-active compounds (32). Indeed, the absence of the *asr* complex in some of the *Halanaerobium* strains isolated in this study (Fig. 2) suggests the potential for niche differentiation in the fractured shales, with other *asr*-containing *Halanaerobium* strains able to potentially utilize sulfite for additional disposal of reducing equivalents. Overall, reactions catalyzed by rhodanese and Asr likely represent an additional mechanism for reductive sulfur transformations that may occur in addition to, or in place of, canonical sulfate reduction. The larger biogeochemical implications of these competing processes across diverse environments remain to be determined.

In the fractured shale system studied here, sulfide and organic acid production by *Halanaerobium* bacteria can potentially drive a series of deleterious processes in the deep subsurface. While sulfide itself is a toxic product, both organic acids and sulfides can contribute to corrosion in the steel infrastructure (11, 24). Sulfide may also react with certain commonly used biocides, limiting their efficiency (33). In addition, sulfides readily complex with metal cations to form sparingly soluble precipitates that may drive decreases in permeability in newly fractured environments (5). Current industry methods for detecting sulfide-producing potential involve the incubation of produced waters in sulfate-containing medium that subsequently turns black (from precipitation of FeS particles) when active sulfate reduction occurs (34). Here, we suggest that sulfide generation in fractured shale environments may be independent of enzymatic sulfate reduction and that sulfide is instead formed through microbial thiosulfate transformations that would go undetected when sulfate medium incubations are used. The availability of thiosulfate from either chemical additives or natural rock leaching, coupled with the seemingly ubiquitous presence of *Halanaerobium* bacteria across hydraulically fractured shales, suggests that sulfide generation is likely to occur across the majority of these environments. New diagnostic tests for sulfidogenesis via thiosulfate reduction in fractured shales are encouraged to accurately identify the prevalence of this metabolism in the deep subsurface.

## MATERIALS AND METHODS

### Fluid sampling.

Fluids were sampled from a Utica Shale well located in eastern Ohio in 2014 and 2015. Input fluids, consisting of recycled produced fluids and lake water, were sampled from a well pad holding tank. Input fluids were aerobically sampled from the tank into sterile high-density polyethylene (Nalgene) bottles and characterized in the lab. Anaerobic produced fluids were recovered at the gas-water separator and stored anaerobically at 4°C. The gas-fluid separator could hold ~5,560 liters and generally contained half gas and half produced fluids. The flow rate through this separator ranged from 380,000 to 190,000 liters/day, depending on the sampling time point. These flow rates allow for 8 h to be the estimated maximum residence time of produced fluids in the gas-fluid separator. Samples for chemical analyses were anaerobically filtered through a 0.22-µm filter (ion chromatography), or a 0.45-μm isopore polycarbonate membrane filter (sulfur isotopes).

### Thiosulfate measurements.

Thiosulfate was measured with a Dionex ICS 2100 ion chromatograph. Thiosulfate concentrations were quantified by comparing the peak area of standard solutions to samples. Produced fluids and culture samples were diluted 50- to 1,000-fold before analysis because of the high salt content of samples.

### Sulfur isotopes.

Aqueous samples were precipitated as BaSO_4_ powder in accordance with USGS RSIL lab code 1951 (35). Both sulfate and sulfonate (from thiosulfate) present in the aqueous samples could react with BaCl to form BaSO_4_ as a solid precipitate. The solid BaSO_4_ precipitate was sent to the University of Arizona Environmental Stable Isotope Facility for analysis of δ^34^S_SO4_ on an elemental analyzer coupled to a Finnigan Delta Plus mass spectrometer. Reproducibility and accuracy were monitored by duplicate analysis of samples and internal lab standards, previously calibrated to international standards, and were better than 0.2‰ for δ^34^S. All δ^34^S isotope values are reported in per mille relative to the Vienna Cañon Diablo meteorite international standard.

### Metagenomic sequencing and assembly.

Samples of 300 to 1,000 ml were concentrated with 0.22-µm-pore-size polyethersulfone filters (Nalgene; Fisher Scientific). Total nucleic acids were extracted from input and produced fluids by a modified phenol-chloroform nucleic acid extraction method. Libraries were prepared with the Nextera XT Library System in accordance with the manufacturer’s instructions. Sequencing adapters were ligated and library fragments were amplified by 12 cycles of PCR before quantification with the KAPA Biosystems next-generation sequencing library quantitative PCR kit. Following library preparation with a TruSeq paired-end cluster kit (v4), sequencing was performed on the Illumina HiSeq2500 sequencer with HiSeq TruSeq SBS sequencing kits (v4) following a 2 × 150 indexed run recipe. We obtained between 14,899,239 and 43,887,735 reads prior to assembly and between 44,472 and 1,217,104 after quality control trimming (Table S5). Fastq files were generated with CASSAVA 1.8.2. Illumina sequences from each sample were first trimmed from both the 5′ and 3′ ends with Sickle (https://github.com/najoshi/sickle), and then each sample was assembled individually with IDBA-UD (iterative De Bruijn graph de novo assembler for short-read sequencing data with highly uneven sequencing depth) (36, 37) by using default parameters. Unassembled Illumina reads from input and produced fluids were reconstructed into nearly full-length 16S rRNA gene sequences with EMIRGE (38). Trimmed paired-end reads, where both reads were at least 20 nucleotides, were used as inputs with 50 iterations. EMIRGE sequences were chimera checked before phylogenetic gene analyses. Coverage was calculated by mapping reads back to the assemblies with Bowtie2 (39). In order to reconstruct the dominant *Halanaerobium* 5U2 and 6U2 genomes, subassemblies were prepared with the day 96 and 140 samples, respectively, with 1% of the reads. Additional assembly and annotation methods are outlined in detail, with all commands provided, in reference 3.

10.1128/mSphereDirect.00257-17.7 TABLE S5 Total number of reads from each metagenome before and after quality checks. Download TABLE S5, XLSX file, 0.02 MB. Copyright © 2017 Booker et al. 2017 Booker et al. This content is distributed under the terms of the Creative Commons Attribution 4.0 International license.

### Metagenomic binning, annotation, and phylogenetic analyses.

Scaffolds of ≥5 kb were included when binning genomes from the 1% metagenomic assembly. Scaffolds were annotated as described previously (3, 36, 37) by predicting open reading frames with MetaProdigal (40). Sequences were compared with USEARCH (41) to KEGG, UniRef90, and InterProScan (42) with single and reverse best hit (RBH) matches of >60 bases reported. As described by Daly et al. (3), the collected annotations for a protein were ranked A to E. Briefly, reciprocal best BLAST hits (RBH) with a bit score of >350 were given the highest rank (A), followed by reciprocal best BLAST hits to Uniref with a bit score of >350 (rank B), BLAST hits to KEGG with a bit score of >60 (rank C), and BLAST hits to UniRef90 with a bit score of >60 (rank C). Proteins that only had InterProScan matches followed (rank D), while the lowest rank (E) comprised the hypothetical proteins, with only a prediction from Prodigal but a bit score of <60.

We obtained two representative *Halanaerobium* genome resolved “bins” from day 96 and 140 samples by using a combination of phylogenetic signal, coverage, and GC content (36, 37). For each bin, genome completion was estimated on the basis of the presence of core gene sets (highly conserved genes that occur in single copy) for *Bacteria* (31 genes) and *Archaea* (104 genes) with Amphora2 (43). Overages (>1 gene copy/bin) indicating potential misbins, along with GC and phylogeny, were used to manually remove potential contamination from the bins. Both *Halanaerobium* genome bins were >95% complete, with no overages.

Phylogenetic placement of the Utica *Halanaerobium* genomes reconstructed here was determined by using a concatenated ribosomal tree (Fig. 2) of 16 ribosomal proteins chosen as single-copy phylogenetic marker genes (RpL2, RpL3, RpL4, RpL5, RpL6, RpL14, RpL15, RpL16, RpL18, RpL22, RpL24, RpS3, RpS8, RpS10, RpS17, and RpS19). Additional sequences were mined from sequenced genomes within the order *Clostridiales* from the NCBI and JGI IMG databases (March 2017). Each individual protein data set was aligned with muscle 3.8.31 and then manually curated to remove end gaps (44). Alignments were concatenated to form a 16-gene, 40-taxon alignment and then run through ProtPipeliner, a python script developed for phylogenetic tree generation (45). Briefly, alignments are curated with minimal editing by Gblocks (46), model selection is conducted via ProtTest 3.4 (47), and a maximum-likelihood phylogeny for the concatenated alignment is generated with RAxML version 8.3.1 by using 100 bootstrap replicates (48). The resulting phylogenetic tree is visualized in iTOL (49). The alignment generated here is provided as Data Set S1.

10.1128/mSphereDirect.00257-17.8 DATA SET S1 Alignment of concatenated ribosomal proteins used to construct a *Halanaerobium* phylogenetic tree. Download DATA SET S1, TXT file, 0.1 MB. Copyright © 2017 Booker et al. 2017 Booker et al. This content is distributed under the terms of the Creative Commons Attribution 4.0 International license.

### Metabolic profiling.

Known rhodanese and anaerobic sulfite reductase amino acid sequences previously identified in other organisms, including *Halanaerobium* bacteria (17), were queried to each metagenome, genome bin, and sequenced isolate with BLASTp. Homologs (cutoff bit score of >200 and >45.9% identity) and copy number per sample were calculated on the basis of the number of homologues recovered in each sample and are included in Table S2. All of the fasta files used in these queries and recovered from metagenomes are available in Data Set S2.

10.1128/mSphereDirect.00257-17.9 DATA SET S2 Rhodanese and anaerobic sulfite reductase amino acid sequences identified in each metagenome, genome bin, and sequenced *Halanaerobium* isolates. Download DATA SET S2, TXT file, 0.1 MB. Copyright © 2017 Booker et al. 2017 Booker et al. This content is distributed under the terms of the Creative Commons Attribution 4.0 International license.

The distribution of rhodanese and anaerobic sulfite reductase genes across bacterial isolate genomes was assessed by using the JGI IMG database (https://img.jgi.doe.gov/). Since the genus *Halanaerobium* is in the class *Clostridia*, these genomes were searched for the presence of rhodanese-encoding and *asr* genes. There were 1,668 isolate genomes in the JGI IMG database identified in the class *Clostridia* as of May 2017. Each *Clostridia* genome was searched for the presence of *asr* and rhodanese-encoding genes, which resulted in the identification of 728 *Clostridia* isolates with genes for both enzymes. To identify the presence of these genes in other bacterial taxa, 500 random genome isolates from *Alphaproteobacteria*, Deltaproteobacteria, *Bacilli*, *Fusobacteriia*, and the phylum *Cyanobacteria* were searched. No anaerobic sulfite reductase subunits were found in *Alphaproteobacteria*, *Bacilli*, and *Cyanobacteria*. However, Deltaproteobacteria and *Fusobacteriia* did contain organisms that contain both rhodanese-encoding and *asr* genes, so this co-occurrence is not restricted to the class *Clostridia*.

### *Halanaerobium* isolation and laboratory growth.

*Halanaerobium* bacteria were isolated by using produced fluids from a Utica Shale well that were streaked onto anaerobic yeast extract-peptone-dextrose (YPD) medium (ATCC medium 1245). Resulting colonies were picked and transferred to liquid YPD medium before checking for purity via microscopy and 16S rRNA gene sequencing. Subsequently, isolates (including WG8) were grown in defined saltwater liquid medium containing NH_4_Cl at 1.0 g/liter, MgCl_2_ ⋅ 6H_2_O at 10 g/liter, CaCl_2_ ⋅ 2H_2_O at 0.1 g/liter, KCl at 1.0 g/liter, NaCl at 100 g/liter, cysteine-HCl at 0.72 g/liter, 10 ml of mineral solution, and 10 ml of vitamin solution (50). This medium was amended with Na_2_S_2_O_3_ ⋅ 5H_2_O at 10 mM, d-glucose at 10 mM, K_2_HPO_4_/KH_2_PO_4_ at 500 μM, and 0.2% NaHCO_3_ after autoclaving. All components were degassed with 99.9% N_2_ to remove trace dissolved oxygen. All *Halanaerobium* isolates were routinely cultured in this anaerobic medium at 40°C in the dark. *Halanaerobium* WG8 growth rates were determined with two sets of triplicate anaerobic 10-ml cultures, with one triplicate set amended with 10 mM Na_2_S_2_O_3_ ⋅ 5H_2_O. Biomass growth was quantified by measuring optical density at 600 nm over the course of 10 days, with growth rates calculated during the period of exponential growth.

### Sulfide measurements.

Sulfide in produced water samples was measured with the Hach colorimetric methylene blue assay. Sulfide concentrations were quantified by comparing the sample absorbance values to those of a standard curve. For laboratory experiments to assess microbial sulfide production, the elevated thiosulfate concentrations interfered with the Hach methylene blue assay, instead producing a cloudy precipitate that prohibited absorbance reading. Instead, a modified zinc acetate sulfide trap approach was used to quantify sulfide production. *Halanaerobium* WG8 was incubated in liquid medium described above with alterations of Na_2_S_2_O_3_ ⋅ 5H_2_O at 1 mM and no cysteine-HCl. Cells in this medium were incubated for 10 days at 40°C in the dark. Sulfide production was tracked by using 72 individual anaerobic *Halanaerobium* enrichments set up with the liquid medium described above. Control experiments contained heat-killed *Halanaerobium* cells. Gaseous sulfide production was measured by using internal open sulfide traps containing 2.5 ml of anoxic 10% zinc acetate modified from reference 51. Sulfide production was measured six times over the 10-day experiment, with sampling at each time point performed in sextuplicate. Twenty-four hours prior to each sulfide production checkpoint, enrichments were shaken at 150 rpm to encourage gaseous sulfide production. Hydrochloric acid was not used to drive aqueous sulfide into the gaseous phase because of issues with abiotic sulfide generation in both positive and negative controls. Our measured values represent only gaseous hydrogen sulfide and therefore underestimate the true sulfide concentration generated in these experiments. To estimate the percent recovery of sulfide in the zinc acetate traps, known concentrations of sulfide were used to spike standards, which were incubated for 24 h while shaking at 150 rpm. These standards were prepared in triplicate and used to calculate a percent sulfide recovery of 39% ± 5%. Following shaking, sulfide traps were removed and the 10% zinc acetate solution was analyzed for zinc sulfide with the Hach methylene blue assay and measured at 665 nm.

### Comparative proteomic sample preparation.

Total protein profiles of *Halanaerobium* WG8 grown in the presence or absence of thiosulfate were determined by shotgun proteomics. Triplicate biomass from previously described growth experiments was harvested at mid-log phase by centrifugation at 10,000 rpm and 4°C for 10 min. The cell pellets were immediately flash frozen in liquid nitrogen to preserve protein signatures. Frozen pellets were stored at −80°C until shipment on dry ice to the Pacific Northwest National Laboratory for analysis. For total protein, each cell pellet was thawed and underwent centrifugation at 2,500 × *g* for 5 min. The supernatant was then removed and snap frozen in liquid nitrogen for storage at −80°C. To each cell pellet, 100 µl of UPX Universal Protein Extraction buffer (Expedeon, San Diego, CA) was added and water bath sonicated into solution. Each sample was incubated at 95°C for 5 min to ensure cell lysis, as well as reduction and solubilization of proteins. The samples were then vortexed and sonicated for 2 min, lightly spun to collect condensate, and allowed to cool at 4°C for 45 min. The samples were then centrifuged at 15,000 × *g* for 10 min, and filter-aided sample preparation (FASP) (52) kits were used for protein digestion (Expedeon, San Diego, CA) in accordance with the manufacturer’s instructions. Briefly, 400 µl of 8 M urea (all reagents included in the kit) was added to each 500-µl FASP spin column (30,000 molecular weight cutoff), up to 100 µl of the sample in UPX buffer was added, and the column was centrifuged at 14,000 × *g* for 30 min to bring the sample all the way to the dead volume. The waste was removed from the bottom of the tube, and another 400 µl of 8 M urea was added to the column, which was centrifuged again at 14,000 × *g* for 30 min; this process was repeated once more. A 400-µl volume of 50 mM ammonium bicarbonate (provided) was added to each column, which was centrifuged for 20 min; this was done twice. The column was placed into a new fresh, clean, and labeled collection tube. Digestion solution was made by dissolving 4 μg of trypsin in 75 μl of 50 mM ammonium bicarbonate solution and added to the sample. Each sample was incubated for 3 h at 37°C with shaking at 800 rpm on a ThermoMixer with a ThermoTop (Eppendorf, Hamburg, Germany) to reduce condensation in the cap. A 40-µl volume of ammonium bicarbonate solution was added to the resultant peptides, and then they were then centrifuged through the filter and into the collection tube at 14,000 × *g* for 15 min. Another 40 µl of ammonium bicarbonate solution was added to the filter and then centrifuged through it. The peptides were concentrated to ~30 µl with a SpeedVac. Final peptide concentrations were determined with a bicinchoninic acid assay (Thermo Scientific, Waltham, MA). Each sample was diluted to 0.3 µg/µl and placed into a vial for mass spectrometry (MS) analysis.

### Comparative proteomic measurements.

Peptides mixtures were initially separated with a two-dimensional liquid chromatography (LC) ACQUITY ultraperformance LC M-class system (Waters, Milford, MA) with a fused-silica hand-packed column (75-µm inside diameter, 70 cm long) with 3-µm particle Jupiter C_18_ derivatized silica beads (Phenomenex, Torrance, CA). Mobile phases consisted of 0 to 100% acetonitrile–0.1% formic acid against water–0.1% formic acid over 100 min. This LC system was coupled to a nanoelectrospray apparatus built in house. MS analyses were performed with a QExactive Pro (Thermo Fisher, Framingham, MA) collecting high-resolution MS (nominal resolution, 35 K) survey scans from 400 to 2,000 *m*/*z* and up to 10 data-dependent high-resolution higher-energy collisional dissociation tandem MS (nominal resolution, 17.5 K) spectra with a 30-s rolling dynamic exclusion period to reduce multiple samplings of the same precursor. Measured peptides were searched against predicted peptides derived from the *Halanaerobium* WG8 genome. Resulting peptide identifications were filtered via MS-GF+ (software used to analyze tandem mass spectra data [53]), *Q* value of ≤0.01, which is an ~1% false discovery rate (FDR) at each individual data set level, as assessed from the decoy identifications. There were 713 reversed identifications (IDs) out of 74,686 total filter passing IDs, for a 0.95% FDR at the peptide-to-spectrum match level. For comparative analyses of triplicate biological replicates, protein spectral counts were normalized by the normalized spectral abundance frequency method (54) and *Z*-score (also called the standard row function) values were calculated. *Z* scores were calculated by subtracting the individual protein abundance from the mean protein abundance across all conditions and dividing the result by the standard deviation of the values. *Z*-score values can be used to determine proteins showing significant changes from their average values. In this study, *Z*-score values were considered significantly different if the difference was ≥2.

### Proton NMR measurements of fermentation products.

Metabolite concentrations were quantified by ^1^H NMR analysis. The one-dimensional (1D) ^1^H NMR spectra of all samples were collected in accordance with standard Chenomx (Edmonton, Alberta, Canada) sample preparation and data collection guidelines (55). Triplicate biological data were acquired on a Varian Direct Drive (VNMRS) 600-MHz spectrometer (Agilent Technologies) equipped with a Dell Precision T3500 Linux workstation running VNMRJ 3.2. The spectrometer system was outfitted with a Varian triple-resonance salt-tolerant cold probe with a cold carbon preamplifier. A Varian standard 1D proton nuclear Overhauser effect spectroscopy (NOESY) with presaturation (TNNOESY) was collected on each sample by the Chenomx standard data collection protocol, which consists of a nonselective 90º excitation pulse, a 100-ms mixing time, an acquisition time of 4 s, a spectral width of 12 ppm, and a temperature control setting of 25°C. A presaturation delay of 1.5 s was used to optimize water suppression. Collected spectra were analyzed with Chenomx 8.1 software (Edmonton, Alberta, Canada) with quantifications based on spectral intensities relative to a calibrated reference solution (100% D_2_O, 0.5 mM 2,2-dimethyl-2-silapentane-5-sulfonate-d6 sodium salt), similar to a previously described protocol (3).

### Thiosulfate shale leaching.

Thiosulfate produced from water-rock interaction with Utica Shale samples was determined via two leaching experiments. For both experiments, shale samples were gently crushed with a mortar and pestle, and then approximately 0.5 g of rock powder was added to a 50-ml polypropylene centrifuge tube with 50 ml of MilliQ water. In the first set of experiments, the supernatant was removed and replaced with fresh water three times, with the duration of each leaching step increasing with the subsequent water addition (1 day, 2 days, 2 weeks, 3 weeks). In the second set of experiments, the shale was allowed to react with water for approximately 7 months before the supernatant was removed. Supernatant samples were filtered and analyzed for anions by ion chromatography as described previously.

### Accession number(s).

All of the metagenomic nucleotide files used in this study are publicly available in the JGI Genome Portal database (http://genome.jgi.doe.gov/) under IMG ID numbers 3300006782, 3300006633, 3300007158, 3300006632, 3300006807, 3300006866, 3300006630, 3300006781, 3300006629, 3300006780, 3300007157, and 3300006798.
