# Supplementary figures and images for: Sulfide Generation by Dominant Halanaerobium Microorganisms in Hydraulically Fractured Shales
Source: mSphere. 2017 Jul 5;2(4):e00257-17. doi: 10.1128/mSphereDirect.00257-17 (PMC5497025; doi:10.1128/mSphereDirect.00257-17)

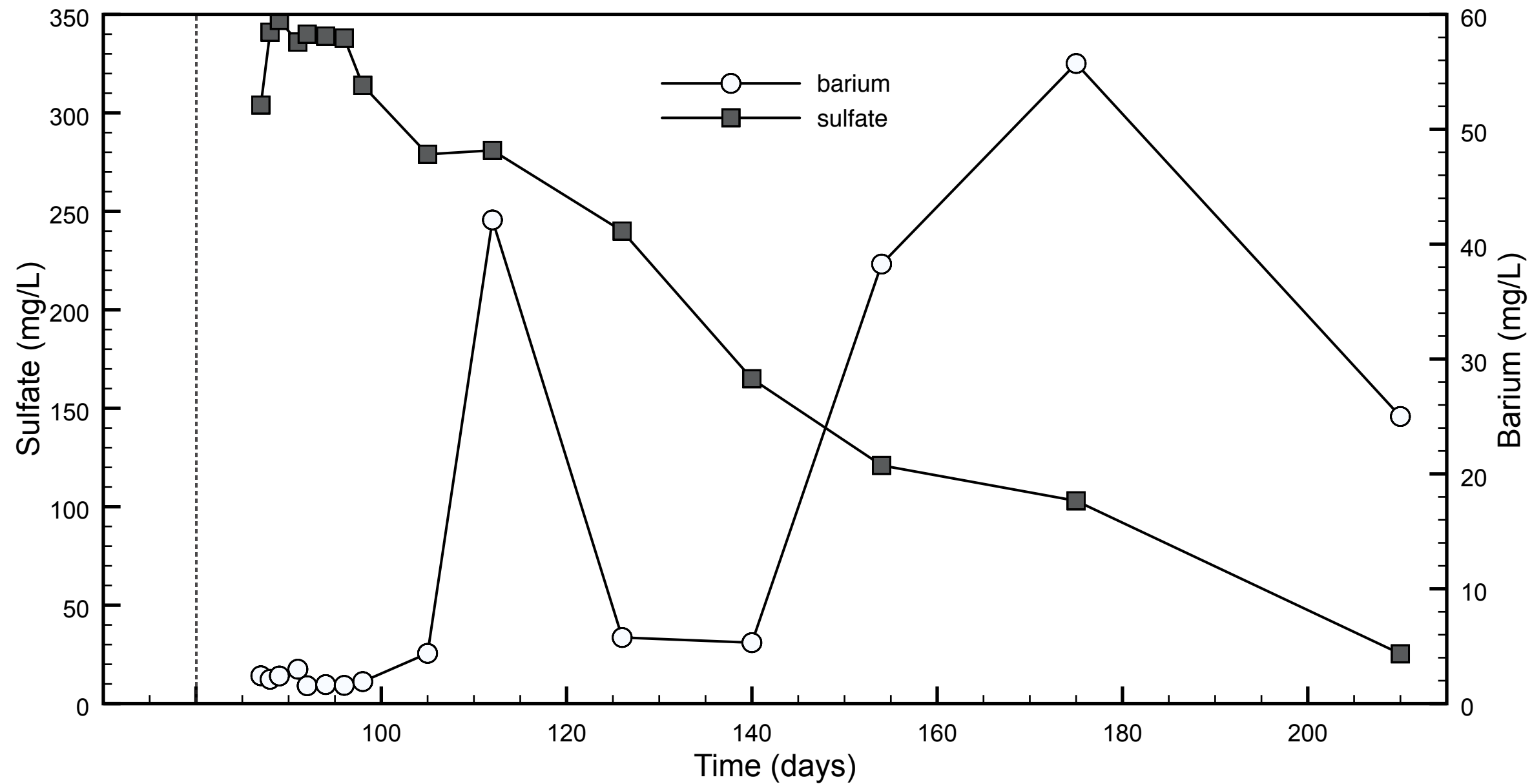

Supplement: FIG S1 [file sph004172314sf1.pdf]

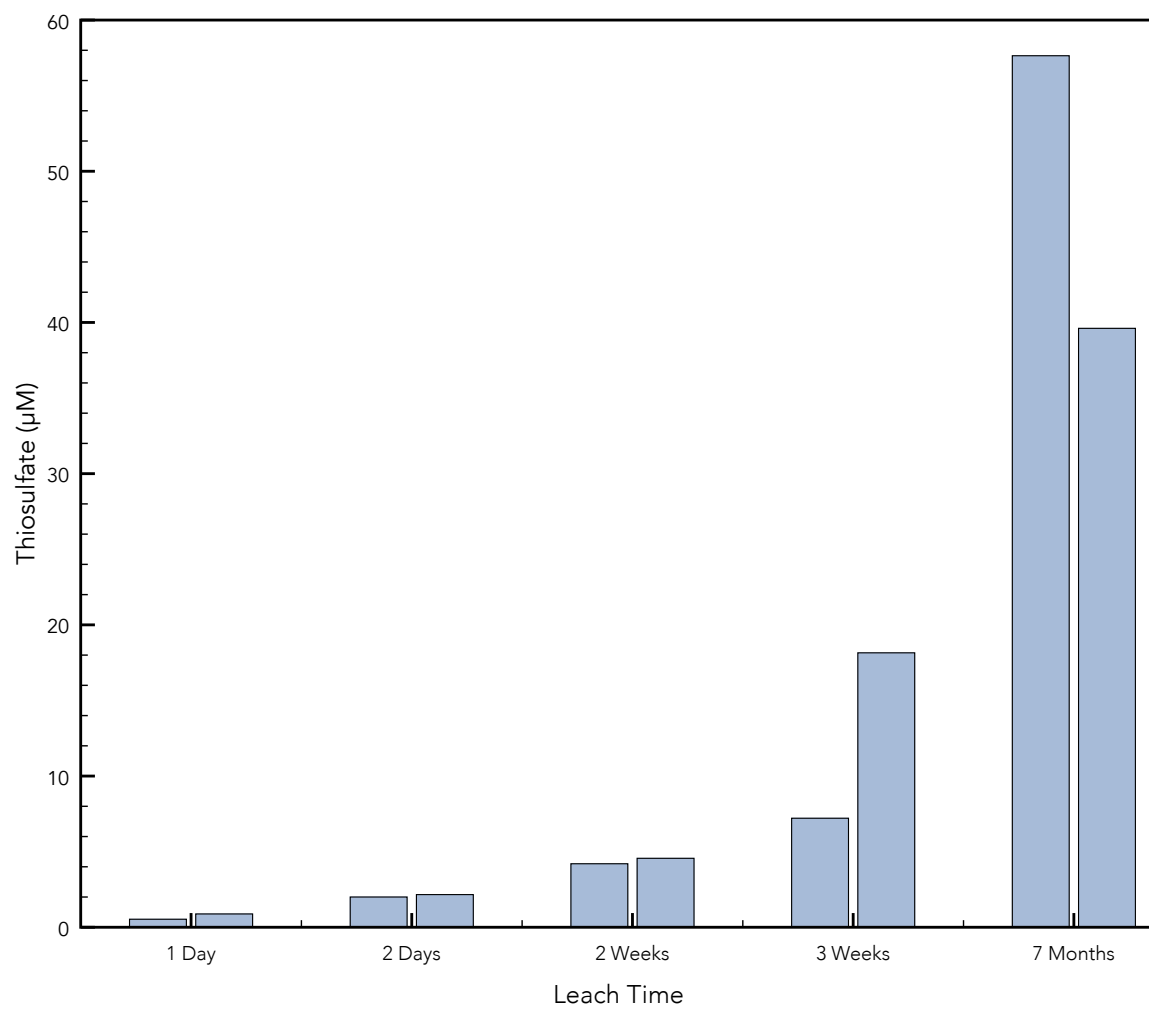

Supplement: FIG S2 [file sph004172314sf2.pdf]
